# Diversification and selection pattern of *CYP6B* genes in Japanese *Papilio* butterflies and their association with host plant spectra

**DOI:** 10.7717/peerj.10625

**Published:** 2020-12-22

**Authors:** Ai Sato, Yu Okamura, Masashi Murakami

**Affiliations:** 1 Community Ecology Lab, Faculty of Science, Chiba University, Chiba, Japan; 2 Department of Entomology, Max Planck Institute for Chemical Ecology, Jena, Germany

**Keywords:** Plant, Counteradaptation, Secondary metabolites, Herbivorous insect, *Papilio*

## Abstract

Herbivorous insects are thought to have evolved counteradaptations to conquer chemical defenses in their host plants in a stepwise co-evolutionary process. *Papilio* butterflies use *CYP6B* gene family members to metabolize furanocoumarins in their Rutaceae or Apiaceae host plants. CYP6Bs have functionally diverged among *Papilio* species to be able to metabolite diverse types of furanocoumarins in their host plants. In this study, we examined the diversification and selection patterns of *CYP6B* among nine *Papilio* species in Japan (eight Rutaceae specialists and one Apiaceae specialist) and their association with host plant spectra and furanocoumarin profiles. We compared host plant spectrum of eight Rutaceae feeding *Papilio* species and also performed a furanocoumarin profiling of their host plants. In addition, we reconstructed *CYP6B* gene phylogeny and performed selection analysis based on the transcriptome data of those nine *Papilio* species. Among Rutaceae-feeding *Papilio* species, host plant spectrum differences were correlated with their furanocoumarin profiles. However, all tested *Papilio* species had similar duplicated sets of *CYP6B*, with no apparent lineage-specific or host plant-specific pattern of *CYP6B* diversification. Selection analysis showed a signature of positive selection on a *CYP6B* branch. The positively selected sites located at predicted substrate recognition sites and we also found that these *CYP6B* genes were observed only in Rutaceae-feeding species. These findings indicate that most *CYP6B* diversification occurred in ancestral species of these *Papilio* species, possibly in association with specific host plant chemical defenses and subsequent gene loss due to host specialization. These processes would have shaped the complex diversification patterns of the *CYP6B* gene family in *Papilio* butterflies. Our results also show potentially important* CYP6B* clades among *Papilio* species which likely to have diverged functions and associated with host plant phytochemicals in ancestral *Papilio* species.

## Introduction

Insect herbivores have evolved counteradaptations to overcome diverse chemical defenses in their host plants. A series of reciprocal evolution events between these counteradaptations and plant chemical defenses is thought to mediate the diversification of both herbivores and plants (Ehrlich & Raven, 1964). Several studies have revealed the molecular or genetic bases of those counteradaptations, such as glucosinolate sulfatases found in *Plutella xylostella* against glucosinolate based-defenses in their Brassicaceae host plants (Ratzka et al., 2002) or UDP-glycosyltransferases in Heliothine moths acting to overcome gossypols produced by cotton plants (Krempl et al., 2016).

*Papilio* butterflies use *CYP6B* gene family members to metabolize furanocoumarins, which are among the major secondary metabolites in Rutaceae or Apiaceae host plants (Cohen, Schuler & Berenbaum, 1992). To date, more than 50 chemically diverse furanocoumarins have been identified (Peroutka et al., 2007); most of these are toxic as they bind to DNA or proteins upon activation by ultraviolet light (Berenbaum, 1995). The induction profiles and functional divergence of *CYP6B* genes against different types of furanocoumarins have been evaluated in North American *Papilio* species (Cohen, Schuler & Berenbaum, 1992; Li et al., 2004; Wen et al., 2006). Some *CYP6B* gene copies have different substrate specificities or functional efficiencies against various furanocoumarins. For example, a *CYP6B* gene found in *Papilio polyxenes*, which is a specialist consumer of furanocoumarin-containing plants, shows higher activity against a specific type of furanocoumarins than does an analogous gene in *Papilio canadensis*, which is unlikely to encounter furanocoumarins (Li, Schuler & Berenbaum, 2003). In addition, even in closely related *Papilio* species, CYP6B induction profiles and functions can be different, highlighting their host plant differences (Li, Berenbaum & Schuler, 2001). These findings demonstrate that *CYP6B* gene family members experienced diversification and subsequent functional divergence in *Papilio* species, potentially in association with the furanocoumarin profiles of their host plants.

Identifying patterns of gene duplication and selection is key to understating the evolutionary steps through which organisms adapt to novel or changing environments (Ohno, 1970; Zhang, 2003). Several studies have shown that gene duplication may have a strong impact on the ability of herbivorous insects to acquire a novel detoxification capability to overcome diverse secondary metabolites in their host plants. In *Plutella xylostella*, gene duplication and subsequent functionalization of glucosinolate sulfatases allow the species to overcome a broader range of glucosinolates in their hosts (Heidel-Fischer et al., 2019). Furthermore, counteradaptation of Pieridae butterflies to glucosinolates, nitrile specifier proteins (NSPs), results in a gene birth–death dynamics in tandem with the diversification of glucosinolates in host plans (Wheat et al., 2007; Fischer et al., 2008; Edger et al., 2015). These genes involved in host plant adaptation are thought to be under strong selection pressure after they acquire novel detoxification functions; such evidence of positive selection has been observed in both glucosinolate sulfatases in *P. xylostella* and *NSP*s in pierid butterflies (Heidel-Fischer et al., 2019; Okamura et al., 2019). Although several studies have suggested that *CYP6B* diversification and functionalization are also important for *Papilio* species to overcome diverse furanocoumarins in their host plants (Cohen, Schuler & Berenbaum, 1992; Berenbaum, Favret & Schuler, 1996; Li et al., 2004; Wen et al., 2006), the patterns of diversification and the evolutionary forces acting on *CYP6B* gene family members and their association with host plant furanocoumarin profiles remain unclear.

In this study, we examined nine *Papilio* species in Japan to identify patterns of *CYP6B* diversification and selection associated with the spectra of the host plants of these species. Eight of these *Papilio* species are Rutaceae specialists, and the remaining species feeds exclusively on Apiaceae plants. Among the Rutaceae specialists, their host plants partially overlapped. However, *P. memnon* and *P. polytes* frequently use citrus plants, whereas *P. macilentus* uses *Orixa* spp*. P. macckii* uses *Phellodendron amurense*, and *P. dehaanii* uses *Zanthoxylium* spp. more frequently, whereas *P. machaon* has shifted to exclusive use of Apiaceae, although its sister species *P. xuthus* uses Rutaceae.

Previous studies have shown that the CYP6B in a species has different substrate specificities associated with differences in the furanocoumarin profile of its host plants (Li, Berenbaum & Schuler, 2001; Li, Schuler & Berenbaum, 2003). Thus, each *Papilio* species appears to have specifically evolved *CYP6B* genes adaptive to the furanocoumarin profile of its host plants. Since differences in host plant spectra among *Papilio* species have not been well evaluated in the context of host furanocoumarin profile data, we analyzed host plant furanocoumarin profiles to determine whether the host plant spectrum of each *Papilio* species was associated with furanocoumarin profile differences.

Next, we assessed whether the diversification or selection pattern of *CYP6B* was associated with the host plant spectrum (and potentially with the furanocoumarin profile) and/or *Papilio* lineage evolution. Some CYP6B can metabolize only a specific subset of furanocoumarins (Wen et al., 2006). This functional specialization may cause specific *CYP6B* diversification patterns, such as rapid diversification, in *Papilio* species that feed exclusively on specific host plants. In such cases, strong selection pressure on these genes would be expected. We performed transcriptome analyses in all nine *Papilio* species with a phylogenetic analysis of *CYP6B* genes expressed in the larval gut and investigated their specific diversification patterns or signatures of positive selection.

## Materials & Methods

### *Papilio* host plant data

Host plant data for nine *Papilio* species (*P. dehaanii*, *P. maackii*, *P. xuthus*, *P. machaon*, *P. helenus*, *P. memnon*, *P. macilentus*, *P. protenor*, and *P. polytes*) were collected from the public database InsectInDB (http://insect-plant.org). Since there was an inflated number of *Citrus* cultivars or related species in the lists compared to wild Rutaceae plants, the number of host plant of each *Papilio* species were biased when the species feed on *Citrus* plants and did not highlight their diet breadth. Therefore, we performed principal component analysis (PCA) on host plant data from Rutaceae specialist *Papilio* species to identify potential differences among their host plant spectra. The PCA was performed with *prcomp()* function in R software (R Core Team, 2019) to acquire PC1 and PC2 scores as indicators of the host plant spectrum of each *Papilio* species. We excluded *P. machaon* from this analysis because it feeds exclusively on Apiaceae.

### Rutaceae furanocoumarin profiling and comparison with *Papilio* host plant spectra

To investigate the furanocoumarin profiles of Rutaceae plant species, we collected undamaged leaves from 13 Rutaceae plant species from wild and cultivar plants listed as *Papilio* hosts in the host plant database (*Citrus depresa*, *C. junos*, *C. limon*, *C. trifoliata*, *C. unshiu*, *Orixa japonica*, *Phellodendron amurense*, *Skimmia japonica*, *Toddalia asiatica*, *Zanthoxylum ailanthoides*, *Z. armatum*, *Z. piperitum*, and *Z. schinifolium*; http://insect-plant.org). The collected fresh leaves were immediately frozen at −20 °C and freeze-dried for further chemical analysis. We used liquid chromatography–electrospray ionization mass spectrometry to quantify furanocoumarins in the sampled leaves. We ground 20 mg freeze-dried leaves using metal balls in 2-mL tubes and added one mL 80% methanol and 2.5 µM lidocaine as an internal standard. We centrifuged the samples at 9,000 rpm for 3 min and analyzed the collected supernatants. We used a bridged ethyl hybrid (BEH) C18 column (Acquity C18; 1.7 × 2.1 × 100 mm, Waters, Milford MA, USA) for reversed-phase LC and set soluble A as 0.1% HOOH water and soluble B as 0.1% HCOOH acetonitrile. The flow rate was 0.3 mL/min, using a program of 0.5% B (0–1 min), 0.5–80% B (1–12 min), 80–99.5% B (12–15 min), and 99.5–0.5% B (15–20 min). We extracted peaks with m/z ratios of 100–400 and identified furanocoumarins with eight standards (angelicin; CAS 523-50-2, bergapten; CAS 484-20-8, imperatorin; CAS 482-44-0, isobergapten; CAS 482-48-4, isoimperatorin; CAS 482-45-1, isopimpinellin; CAS 482-27-9, psoralen; CAS 66-97-7, and xanthotoxin; CAS 298-81-7). The acquired peaks and retention times of each standard were used to identify and quantify furanocoumarins in the samples. We used the Xcalibur software (Thermo Fisher Scientific, Waltham, MA, USA) to extract peaks and identify furanocoumarins; we removed peaks lower than 0.001. Each plant species was analyzed in triplicate.

We compared the total amount of detected furanocoumarins in each host plant among plant species. Since different furanocoumarins can exert different toxicity against herbivores, we also compared furanocoumarin diversity among plant species using the chemical complexity index (CCI, Becerra, Noge & Venable, 2009); the CCI was calculated based on two Shannon indices of chemical diversity, one based on presence/absence and the other based on relative concentration. We calculated these two Shannon indices based on furanocoumarin profiles of each plant species and CCI was acquired by totaling these two indices (Becerra, Noge & Venable, 2009). For each *Papilio* species, we calculated the average level of detected furanocoumarin and the CCIs of their host plants. We performed linear regression analyses to compare these values with the PC1/2 scores, which showed the host plant spectrum of each *Papilio* species. This analysis allowed us to examine the correlation between differences in *Papilio* host plant spectra and furanocoumarin profiles among host plants.

### Larval sampling for transcriptome analyses

We collected eggs or fertilized female butterflies from wild populations of *Papilio* species in Japan (Table S1). Fertilized females were kept with suitable host plants for egg laying. Hatched neonate larvae were reared with their host plants until the second instar. Five individuals of each *Papilio* species were dissected and their guts were extracted for transcriptome analysis. We extracted RNA using the RNeasy Mini Kit (Qiagen, Hilden, Germany) and pooled one unit of RNA from each of five individuals for sequencing. Genomic DNA in the samples were digested with the TURBO DNA-free Kit (Thermo Fisher Scientific, Waltham, MA, USA). After quality confirmation using the Agilent 2100 Bioanalyzer, samples were used for library preparation. We performed 100-bp pair-end sequencing using the Illumina HiSeq 4000 system.

### De novo transcriptome assembly, *CYP6B* gene extraction, and phylogenetic analysis

We controlled the quality of the raw reads using the Trimmomatic ver. 0.32 command line tool, with the settings LEADING:10, TRAILING:10, SLIDINGWINDOW:4:20, and MINLEN:40 (Bolger, Lohse & Usadel, 2014). After trimming, the read quality was verified with FastQC software then *de novo* assembly was performed with the Trinity ver. 2.1.1 software (Grabherr et al., 2011; Haas et al., 2013). We extracted the longest isoforms from the assembly using the Trinity command “get_longest_isoform_seq_per_trinity_gene.pl” then performed tblastn (Altschul et al., 1990; Camacho et al., 2009) on the assembled contigs using *CYP6B* genes of *Papilio* species from previous studies as queries (accession nos. AAB06742.1, AAB06743.1, AAK69477.1, AAK69478.1, AAK69497.1, AAK69499.1, AAK69500.1, AAK69503.1, AAK69504.1, AAK69494.1, AAK69495.1, AAK69496.1, AAK69498.1, AAK69501.1, AAK69505.1, and AAB06741.1) with the e-values set at 0.0001. After extracting hits from the tblastn search, sequences shorter than 300 bp were eliminated. We translated the extracted contigs to amino acid sequences and aligned them with reference sequences using the mafft tool (Katoh & Standley, 2013), with the –auto option. Lepidopteran CYP sequences found in the P450 database (http://drnelson.uthsc.edu/cytochromeP450.html) were used as additional reference sequences in this analysis. We reconstructed a maximum likelihood (ML) phylogeny using the IQ-TREE software with 1000 bootstrap replicates (Nguyen et al., 2015). Based on the resulting phylogeny, we excluded genes that were not included in the *CYP6B* clade and assigned sequences with >  55% amino acid identity to the reference as *CYP6B* genes. To observe the patterns of *CYP6B* gene duplication and loss among tested *Papilio* species, we conducted gene-tree species-tree reconciliation analyses with NOTUNG software (Stolzer et al., 2012). Lower supported nodes (<80% in bootstrap values) were rearranged and the gene tree were reconciled along with the species tree generated from the transcriptome data (see Species phylogeny reconstruction section below).

To see the relationships between host plant spectrum and *CYP6B* duplication patterns among *Papilio* species, we also analyzed correlations between the observed *CYP6B* numbers and host plant spectrum (PC1/2 scores and average host plant CCI or furanocoumarin amount) of each *Papilio* species by linear regression.

As an *ad hoc* analysis, we also analyzed available genome data from *P. machaon* (NCBI: GCA_001298355) and *P. momnon* (NCBI: GCA_003118335) to confirm the absence of a *CYP6B* gene with a signature of positive selection (see Results). We performed the analyses described above using the assembled genome and extracted *CYP6B* genes. Unaligned intron regions of the extracted genes were trimmed out in the phylogenetic analysis. We performed the same phylogenetic analysis again, including *CYP6B*s extracted from the genomes.

### Species phylogeny reconstruction

BUSCO single-copy gene sets were extracted and used to reconstruct *Papilio* species phylogeny from the transcriptome data by running BUSCO (Seppey, Manni & Zdobnov, 2019) on each transcriptome assembly. When duplicate hits occurred, we extracted the longer contig as the representative. We extracted BUSCO genes that were found in all species, aligned the sequences using the mafft tool, and concatenated the alignment for phylogenetic analyses. The concatenated alignment was de-gapped with using the TrimAl software (Capella-Gutiérrez, Silla-Martínez & Gabaldón, 2009) and IQ-TREE was used to reconstruct ML phylogenies of the nine *Papilio* species from the concatenated sequences, with substitution model selection and 1000 bootstrapping iterations. RNA sequencing data from four pierid butterflies (*P. brassicae*, *P. canidia*, *P. melete*, and *P. napi*) were used as outgroups (EBI Accession numbers: ERX2829492–ERX2829499, ERX3552761).

### Detection of positive selection

We performed branch-site model tests to examine patterns of positive selection among *CYP6B* gene family members in *Papilio* species using the codeml program implemented in the PAML software package (Yang, 2007). We excluded outgroups and reconstructed a ML tree (nucleotide) for *CYP6B* using IQ-TREE. All the major internal branches having >70% node support according to the bootstrap values were tested. We used model 2, with NSsites = 2, and ran an alternative model, which allows varied the non-synonymous substitution (dN) to synonymous substitution (dS) ratios (dN/dS) across sites and lineages, as well as a null model with a fixed dN/dS ratio (fixed_omega = 1). We compared the results of the two models using the likelihood ratio test (LRT) with a chi-square distribution to detect significant differences between the alternative and null models. *P* values were adjusted with false discovery rates. We performed subsequent Bayes empirical Bayes analysis (0.95 cut-off) to identify sites potentially under positive selection once the alternative model was selected by the likelihood ratio test. The gene tree topology with lower node supports might affect the results, therefore, we also performed the same branch-site model tests using a gene tree rearranged and reconciled by NOTUNG along with the species tree. The same branches were selected for the test if the branch was still conserved after NOTUNG gene tree rearrangement and reconciliation processes to confirm the results of branch-site model tests based on the ML tree.

## Results

### Host plant spectrum associated with host plant furanocoumarin profiles

**Figure 1:**
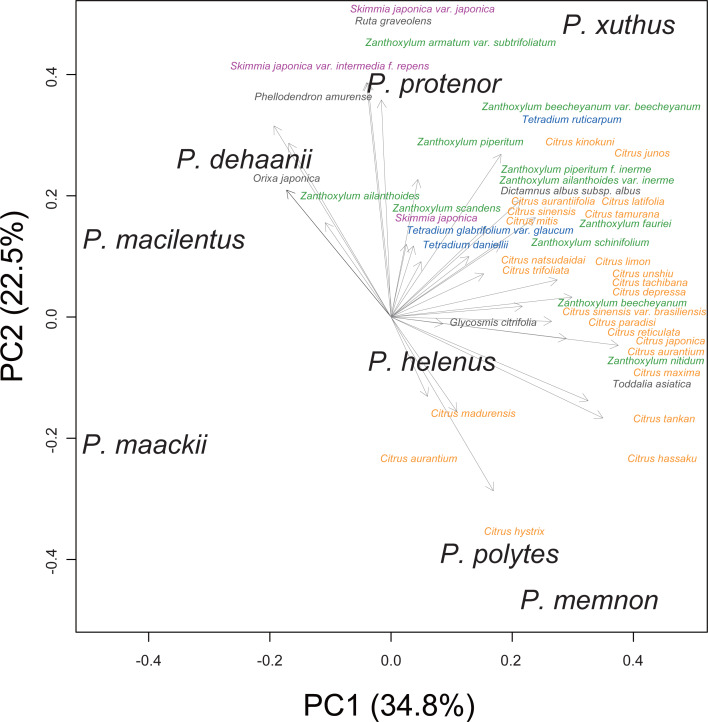
Host plant spectra biplot of eight Rutaceae feeding *Papilio* species. Each *Papilio* species is shown in black letters. Host plants are shown as vectors with gray arrows. Major host plant genera are colored as; orange: *Citrus*, green: *Zanthoxylum*, purple: *Skimmia*, and blue: *Tetradium*. Values in the bracket at each axis are proportion of variances.

We performed principal component analysis to compare the host plant spectra of eight Rutaceae-feeding *Papilio* species (Fig. 1). Although the host plant spectra overlapped among *Papilio* species, *P. memnon* and *P. polytes* tended to use more *Citrus* species, whereas *P. dehaanii*, *P. protenor*, and *P. macilentus* were more reliant on *Orixa japonica*, *Skimmia* spp., or *Zanthoxylum* spp. We used these PC1/2 scores as indicators of the host plant spectra of these *Papilio* species in later analyses.

**Figure 2:**
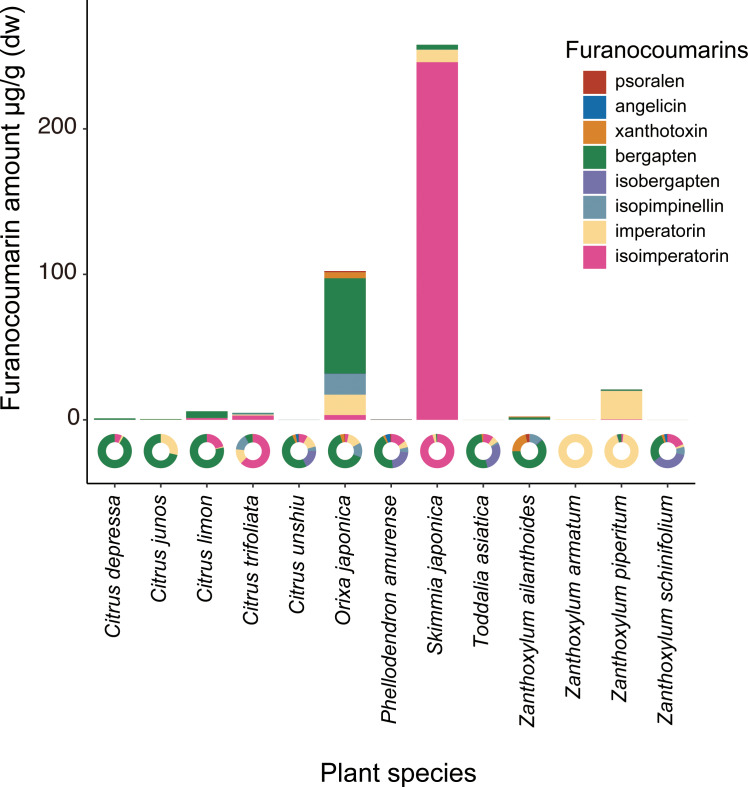
The detected amount and composition of furanocoumarins from Rutaceae leaf samples. The pie charts show furanocoumarin composition of each plant species.

The chemical analysis showed that *Orixa japonica* and *Skimmia japonica* had higher levels of furanocoumarins than those of *Citrus* species (Fig. 2, Table S2). Bergapten was dominant in *Orixa japonica*, and isoimperatorin was dominant in *Skimmia japonica*. These findings were consistent with those of previous studies (Atkinson, Boyd & Grundon, 1974; Zobel & Brown, 1990). We detected lower levels of furanocoumarins in the leaves of *Citrus* spp. than in those of other species. Although higher furanocoumarin levels were observed in *Citrus* peels or juice in previous studies (Barreca et al., 2011; Dugrand et al., 2013), lower levels in *Citrus* leaves were also reported in a previous study (Durand-Hulak et al., 2015). Based on this analysis, we calculated CCIs to assess the furanocoumarin profile complexity of each Rutaceae species. *Orixa japonica* and *Zanthoxylum ailanthoides* had higher CCI values (Table S2).

**Figure 3:**
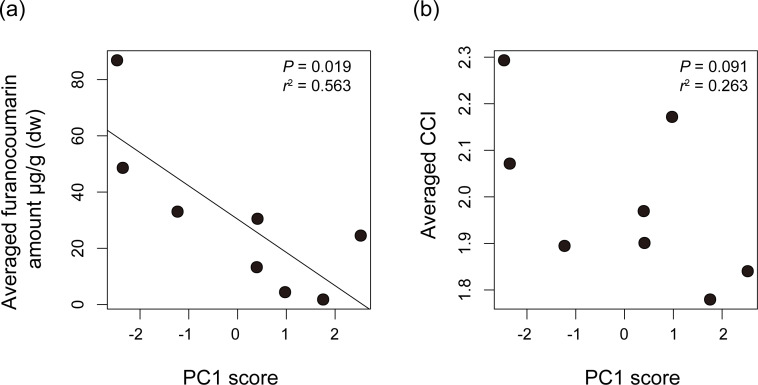
The relationships between host plant spectrum of each *Papilio* species and plant furanocoumarin profiles. The relationships between host plant spectrum of each *Papilio* species (PC1 score) and (A) averaged amount of detected furanocoumarin of their host plants or (B) averaged chemical complexity of furanocoumarin profiles of their host plants. The regression line is shown once it’s significant. *P* values and *R*-square values are shown in each dot plot. The averaged furanocoumarin amount is significantly negatively correlated with PC1 score suggesting that the level of furanocoumarins differed among host plants of each *Papilio* species.

We compared *Papilio* host plant spectra with furanocoumarin profiles of the host plants. The average level of detected furanocoumarin was higher in host plants of *P. macilentus* and *P. maackii* and lower in those of *P. memnon* and *P. polytes* (Table S3). There were no apparent differences in the furanocoumarin CCI among the host plants of each *Papilio* species (ANOVA; *P* = 0.969) (Table S3). We found a significant negative correlation between the average level of detected furanocoumarin in host plants and PC1, which showed the host plant spectrum of each *Papilio* species (Fig. 3A). However, we did not find a significant relationship between the average CCI of the host plants of each *Papilio* species and the PC axes (Fig. 3B). Thus, the level of furanocoumarins but not the complexity differed among host plants of each *Papilio* species.

### No specific diversification patterns of *CYP6B* correlating to patterns of host plant spectrum or species phylogeny

**Figure 4:**
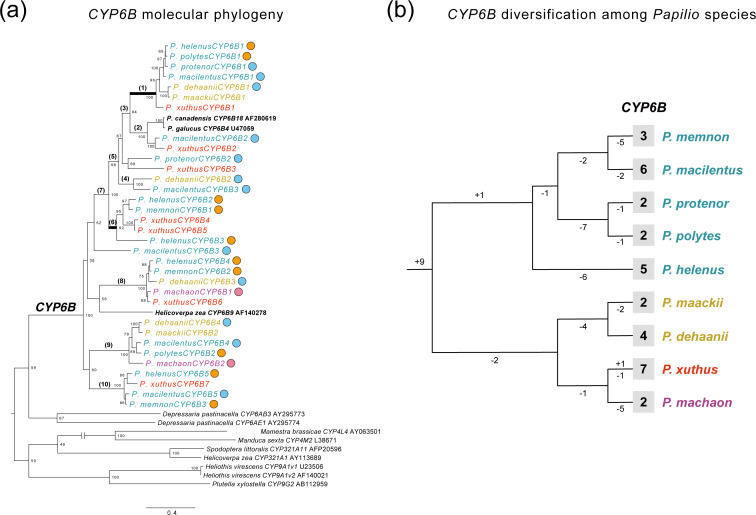
Diversification and selection patterns of *CYP6B*s from nine *Papilio* species. (A) A ML molecular phylogeny of *CYP6B* from transcriptome data of nine *Papilio* species with reference sequences. Each *Papilio* species is colored based on its phylogenetic relationships from reconstructed species tree. Circles with different colors show the primary host plants of each *Papilio* species based on PCA and host plant database (orange: *Citrus*, blue: non-*Citrus* Rutaceae, pink: *Apiaceae*). Numbers on the nodes show bootstrapping values from 1,000 iteration. Branches with bracket are tested with the branch site model for positive selection. The highlighted branches with bold are branches with signature of positive selection. (B) The patterns of *CYP6B* gene duplication and loss among *Papilio* species tested. The species tree is based on transcriptome data (Fig. S1) and each *Papilio* species is colored based on its phylogenetic relationships. The observed numbers of *CYP6B* genes are shown at tip for each species. The numbers on each branch indicate gene duplication and loss events.

The transcriptome assembly statistics are shown in Table S4. We extracted *CYP6B*-related genes from the *de novo* transcriptome assembly of each *Papilio* species and performed phylogenetic analysis. The extracted genes included 33 *CYP6B* genes from nine *Papilio* species. Figure 4A shows an ML phylogeny of *CYP6B* with reference sequences. We also reconstructed a tree of these *Papilio* species using 858 extracted BUSCO genes (Fig. 4B, Fig. S1B) that were found across all transcriptome data and aligned for phylogenetic analysis. The length of this alignment was 567,026 bp after gap elimination.

Generally, we did not detect species- or lineage-specific diversification patterns of *CYP6B* (Fig. 4 and 4B). The gene tree–species tree reconciliation analysis showed that the diversification of *CYP6B* genes among *Papilio* species occurred in the most ancestral *Papilio* species of those tested species, followed by subsequent gene loss in each lineage (Fig. 4B). We detected no apparent diversification pattern associated with the host plant spectra (Fig. 4A). *P. machaon*, which exclusively uses Apiaceae, not Rutaceae, did not exhibit a divergent *CYP6B* clade, which was also observed in the Rutaceae-feeding species. We also compared *CYP6B* gene number of each species and their host plant spectrum and this did not show any significant correlations (Fig. S2).

### Signature of positive selection on *CYP6B* branches among *Papilio* species

The branch-site model tests with both *CYP6B* ML tree and reconciled *CYP6B* tree showed that branch1, which was closer to furanocoumarin-inducible *CYP6B* references (*P. canadensis CYP6B18* and *P. glaucus CYP6B4* in Fig. 4A), displayed a signature of positive selection (Fig. 4A, Fig. S1A, Table 1, Table S5). Branch 1 involved *CYP6B*s from most of the *Papilio* species analyzed, except for *P. machaon* and *P. memnon*. None of the species had duplicated *CYP6B*s associated with this branch in our transcriptome data. At ML tree-based analysis, we found evidence of positive selection on 10 sites at this branch and 8 of them were also found in the same analysis with reconciled *CYP6B* tree (Table 1, Fig. S1). These positively selected sites included substrate recognition sites 3 (SRS3) and SRS6, which may have a strong impact on the substrate specificity of *CYP6B*s (Li, Schuler & Berenbaum, 2003) (Table S5). As an ad hoc analysis, we also searched for these particular *CYP6B* genes within the genomes of *P. machaon* and *P. memnon* and did not find these genes within this particular branch (Fig. S4). At ML tree-based analyses, we also found a signature of positive selection on branch 6 (Fig. 4A, Table 1), which included *CYP6B*s from *P. xuthus, P. helenus,* and *P. memnon*. One site was also located at SRS2 (Fig. S4) and found to be under positive selection at this branch (Table 1). However, the significance at this branch disappeared in the analysis with reconciled tree (Fig. S3).

**Table 1:** Results of branch site model test and positively selected sites based on the ML tree.

Branch	lnL alt	lnL null	delta L	*P* value	*P* value FDR adj	BEB (>0.95)
1	−23390.4	−23394.6	8.31	0.004	0.026^*^	217, 0.962^*^ 223, 0.950^*^ 230, 0.959^*^ 241, 0.955^*^ 262, 0.962^*^ 381, 0.959^*^ 384, 0.959^*^ 417, 0.960^*^ 473, 0.954^*^ 482, 0.954^*^
2	−23404.2	−23404.8	1.18	0.278	0.463	
3	−23404.6	−23406.3	3.24	0.072	0.143	
4	−23410.4	−23410.4	0.00	1.000	1.000	
5	−23406.4	−23406.5	0.21	0.645	0.716	
6	−23404.8	−23408.7	7.80	0.005	0.026^*^	186, 0.969^*^
7	−23403.9	−23406.4	5.05	0.025	0.075	
8	−23406.1	−23406.2	0.23	0.634	0.716	
9	−23406.9	−23409.3	4.72	0.030	0.075	
10	−23405.5	−23405.7	0.41	0.523	0.716	

**Notes.** lnL alt: log likelihood for alternative model which allows having unfixed dN/dS values at the branch. lnL null: log likelihood for null model with fixed dN/dS ratios. Delta L: 2(lnL alt - lnL null) for the likelihood ratio test (LRT). P values are from LRT and adjusted for multiple testing with false discovery rates. BEB analysis shows the site positions with signatures of positive selection with posterior probability (0.95 cutoff). Positions are based on Fig. S3.

* Significance.

## Discussion

In this study, we analyzed the patterns of diversification and selection on *CYP6B* genes associated with host plant spectra or furanocoumarin profiles among *Papilio* species. Even in Rutaceae-specialist *Papilio* species, we observed considerable variation in host plant spectrum patterns. Differences in furanocoumarin concentrations among host plants were correlated with the pattern of larval host use (Fig. 3A). For example, *P. memnon* and *P. polytes* use *Citrus* species as major host plants and our chemical analyses showed that those *Citrus* species tended to have low furanocoumarin levels in their leaves. In contrast, *P. macilentus* and *P. dehaanii* use *Orixa japonica* or *Skimmia japonica*, which had relatively higher furanocoumarin concentrations (Figs. 1 and 2). Since few furanocoumarin standards were commercially available in our chemical analysis, we detected a limited number of furanocoumarins compared with those previously identified (Dugrand-Judek et al., 2015). In addition, there are differences in non-furanocoumarin chemicals among different host plant species. For example, *Zanthoxylum* spp. also contain flavonoids or alkaloids (Javier Patino, Angelica Prieto & Enrique Cuc, 2012), which play defensive roles against herbivores. Although these factors would also affect their host spectrum, our findings indicate that each *Papilio* species has a different ability to overcome furanocoumarins, potentially leading to functional divergence of *CYP6B*s and evolution.

Phylogenetic analysis and gene tree–species tree reconciliation analyses of *CYP6B* genes showed that most of these genes diversified in ancestral *Papilio* species (Fig. 4B). In some herbivorous insects, sequential evolution of genes involved in host plant adaptations occurred to allow the insect to overcome phytochemical diversification in their hosts. In pierid butterflies, birth–death dynamics of *NSP*s has been observed in tandem with the evolution of chemical defenses in their Brassicaceae host plants (Edger et al., 2015). However, most of the *Papilio* species examined in this study had a set of core *CYP6B* genes with no apparent diversification pattern associated with their lineages or host plant spectra (Fig. 4A). Remarkably, we detected no specific diversification patterns of gut expressed *CYP6B*, even in *P. machaon*, which shifted its host completely to Apiaceae plants (Fig. 4A). These findings suggest that the main diversification of *CYP6B* occurred in ancestral *Papilio* species, and that most of these gene sets were conserved even under dynamic host shifts. Although we included nine *Papilio* species in this study, most were from the same subgenus *Papilio Papilio* and are therefore closely related (Wu et al., 2015). Further analysis of a more diverse group of *Papilio* species would shed light on the history of *CYP6B* diversification in a more basal *Papilio* clade, in association with dynamic host shifts, such as host switching, across plant families.

Although we did not detect specific diversification patterns of *CYP6B* among the nine *Papilio* species, our branch-site model test with the ML and reconciled tree found evidence of positive selection in a *CYP6B* branch, branch 1, which was close to the furanocoumarin-inducible *CYP6B* gene clade (Li, Berenbaum & Schuler, 2001). Most of the species tested had transcripts in this branch, suggesting that this gene was acquired by an ancestor of the tested *Papilio* species (Fig. 4A). We also found positively selected sites in SRSs on this branch, which may indicate that this *CYP6B* clade has different substrate specificities. Interestingly, we found that *P. machaon*, an Apiaceae specialist, has lost this gene from its genome (Fig. S4), potentially suggesting that this *CYP6B* is unnecessary for feeding on Apiaceae plants, which likely have different furanocoumarin profiles than Rutaceae plants. Although we did not observe the specific functional activities of this particular *CYP6B* gene against different furanocoumarins or phytochemicals, our results suggest that this *CYP6B* gene is important to understand the evolutionary aspects of chemical interactions between *Papilio* butterflies and their host plants.

In this study, we used short read-based transcriptome analysis to obtain sequences of the *CYP6B* genes expressed in *Papilio* larval guts. Transcriptome analysis is effective for acquiring gene sequences expressed in specific tissues in non-model species. However, this method can overlook candidate genes expressed at lower levels, and distinguishing genes with similar sequences is challenging (Conesa et al., 2016). Additional genome sequences of these *Papilio* butterflies would help us understand the holistic patterns of diversification of *CYP6B*-related genes among *Papilio* species.

## Conclusions

Gene duplication and functional divergence play crucial roles in allowing organisms to adapt to novel environments (Ohno, 1970). In this study, we analyzed patterns of diversification and selection on *CYP6B*-related genes associated with host plant spectra and furanocoumarin profiles among *Papilio* species. Although we detected a correlation between the host plant spectrum of each *Papilio* species and furanocoumarin profiles, we did not observe a clear pattern of *CYP6B* diversification associated with host plant spectra or lineage evolution among these nine *Papilio* species in Japan. However, we found a signature of positive selection on a *CYP6B* branch, which is close to furanocoumarin-inducible *CYP6B* genes in other *Papilio* species identified in previous studies. We also found that this *CYP6B* was lacking in a species exhibiting a shifted host plant spectrum. These results indicate that major *CYP6B* functional divergence occurred in ancestral *Papilio* species, potentially associated with diverse furanocoumarins, and that secondary gene loss may have been caused by shifts in host plant spectra. Our results demonstrate the potential importance of the *CYP6B* clade among *Papilio*. Additional detailed functional characterization of these *CYP6B* genes will be key to understanding how they adapt to plant hosts with diverse furanocoumarins.

##  Supplemental Information

10.7717/peerj.10625/supp-1 Supplemental Information 1 Supplementary Tables and Figures

10.7717/peerj.10625/supp-2 Supplemental Information 2 CYP6B nucleotide sequences

10.7717/peerj.10625/supp-3 Supplemental Information 3 CYP6B protein sequences with references

10.7717/peerj.10625/supp-4 Supplemental Information 4 Concatenated BUSCO gene sequences for species phylogeny reconstruction

10.7717/peerj.10625/supp-5 Supplemental Information 5 Raw furanocoumain quantification data

## Additional Information and Declarations

### Data Availability

The following information was supplied regarding data availability: Data is available at the EBI Sequence Read Archive (SRA): ERS4993295–ERS4993303. The complete study can also be accessed directly at the SRA: PRJEB40013. The extracted CYP gene sequences, extracted BUSCO gene sequences, and the quantified LCMS data from all the samples are available in Supplemental Files.
