# Supplementary material for: Diversification and selection pattern of CYP6B genes in Japanese Papilio butterflies and their association with host plant spectra
Source: PeerJ. 2020 Dec 22;8:e10625. doi: 10.7717/peerj.10625 (PMC7761194; doi:10.7717/peerj.10625)
Supplement: Supplemental Information 1 [file peerj-08-10625-s001.docx]

**Supplementary Tables and Figures**

**Table S1** *Papilio* larvae used for transcriptome analyses

| *Papilio* species | Sampling sites | Host plants for rearing |
| --- | --- | --- |
| *Papilio dehaanii* | 35º 64' N, 139º 28' E | *Zanthoxylum schinifolium* |
|  | 37º 76' N, 140º 58' E | *Orixa japonica* |
|  | 37º 76' N, 140º 58' E | *Orixa japonica* |
|  | 37º 76' N, 140º 58' E | *Orixa japonica* |
|  | 37º 76' N, 140º 58' E | *Orixa japonica* |
| *Papilio helenus* | 34º 96' N, 139º 85' E | *Citrus unshiu* |
|  | 34º 96' N, 139º 85' E | *Zanthoxylum ailanthoides* |
|  | 34º 96' N, 139º 85' E | *Zanthoxylum ailanthoides* |
|  | 34º 96' N, 139º 85' E | *Zanthoxylum ailanthoides* |
|  | 34º 96' N, 139º 85' E | *Zanthoxylum schinifolium* |
| *Papilio maackii* | 42º 67' N, 141º 59' E | *Phellodendron amurense* |
|  | 42º 67' N, 141º 59' E | *Phellodendron amurense* |
|  | 42º 67' N, 141º 59' E | *Phellodendron amurense* |
|  | 42º 67' N, 141º 59' E | *Phellodendron amurense* |
|  | 42º 67' N, 141º 59' E | *Phellodendron amurense* |
| *Papilio machaon* | 34º 96' N, 139º 85' E | *Foeniculum vulgare* |
|  | 36º 34' N, 137º 83' E | *Angelica keiskei* |
|  | 36º 34' N, 137º 83' E | *Angelica keiskei* |
|  | 36º 34' N, 137º 83' E | *Angelica keiskei* |
|  | 36º 34' N, 137º 83' E | *Angelica keiskei* |
| *Papilio macilentus* | 36º 34' N, 137º 83' E | *Zanthoxylum schinifolium* |
|  | 36º 34' N, 137º 83' E | *Zanthoxylum schinifolium* |
|  | 37º 76' N, 140º 58' E | *Orixa japonica* |
|  | 34º 96' N, 139º 85' E | *Zanthoxylum ailanthoides* |
|  | 34º 96' N, 139º 85' E | *Zanthoxylum ailanthoides* |
| *Papilio memnon* | 34º 96' N, 139º 85' E | *Citrus unshiu* |
|  | 34º 96' N, 139º 85' E | *Citrus unshiu* |
|  | 34º 96' N, 139º 85' E | *Citrus unshiu* |
|  | 34º 96' N, 139º 85' E | *Citrus unshiu* |
|  | 34º 96' N, 139º 85' E | *Citrus unshiu* |
| *Papilio polytes* | 24º 45' N, 124º 09' E | *Toddalia asiatica* |
|  | 24º 45' N, 124º 09' E | *Toddalia asiatica* |
|  | 24º 45' N, 124º 09' E | *Toddalia asiatica* |
|  | 24º 45' N, 124º 09' E | *Toddalia asiatica* |
|  | 24º 45' N, 124º 09' E | *Toddalia asiatica* |
| *Papilio protenor* | 35º 62' N, 140º 10' E | *Zanthoxylum piperitum* |
|  | 35º 62' N, 140º 10' E | *Zanthoxylum piperitum* |
|  | 35º 62' N, 140º 10' E | *Zanthoxylum piperitum* |
|  | 35º 62' N, 140º 10' E | *Zanthoxylum piperitum* |
|  | 35º 62' N, 140º 10' E | *Zanthoxylum piperitum* |
| *Papilio xuthus* | 35º 62' N, 140º 10' E | *Citrus unshiu* |
|  | 35º 62' N, 140º 10' E | *Citrus unshiu* |
|  | 36º 34' N, 137º 83' E | *Citrus natsudaidai* |
|  | 36º 34' N, 137º 83' E | *Citrus natsudaidai* |
|  | 36º 34' N, 137º 83' E | *Zanthoxylum schinifolium* |

**Table S2** Detected furanocoumarins amount and calculated CCI in Rutaceae plants (µg/g dw ± SE; *n* = 3). n.d.: not detected

|  | psoralen | | angelicin | | xanthotoxin | | bergapten | | isobergapten | | isopimpinellin | | imperatorin | | isoimperatorin | | CCI |
| --- | --- | --- | --- | --- | --- | --- | --- | --- | --- | --- | --- | --- | --- | --- | --- | --- | --- |
| *Citrus depressa* | n.d. | | n.d. | | n.d. | | 0.9862 | ±0.0943 | n.d. | | n.d. | | 0.0209 | ±0.0095 | 0.0715 | ±0.0181 | 1.261 |
| *Citrus junos* | n.d. | | n.d. | | n.d. | | 0.3991 | ±0.2178 | n.d. | | n.d. | | 0.1605 | ±0.0615 | n.d. | | 1.064 |
| *Citrus limon* | n.d. | | n.d. | | n.d. | | 4.6165 | ±0.1661 | n.d. | | 0.0045 | ±0.0010 | 0.0638 | ±0.0071 | 1.2017 | ±0.0649 | 1.439 |
| *Citrus trifoliata* | 0.0012 | ±0.0002 | 0.0030 | ±0.0012 | 0.0185 | ±0.0002 | 0.3611 | ±0.0336 | 0.0208 | ±0.0018 | 0.7265 | ±0.0363 | 0.6909 | ±0.0303 | 3.0150 | ±0.0587 | 2.131 |
| *Citrus unshiu* | 0.0006 | ±0.0002 | 0.0024 | ±0.0008 | 0.0029 | ±0.0004 | 0.0456 | ±0.0156 | 0.0162 | ±0.0026 | 0.0039 | ±0.0011 | 0.0112 | ±0.0048 | 0.0073 | ±0.0027 | 2.400 |
| *Orixa japonica* | 0.7410 | ±0.0202 | 0.0575 | ±0.0187 | 4.1392 | ±0.2516 | 65.5811 | ±3.3972 | 0.2952 | ±0.1014 | 14.1261 | ±0.6682 | 14.0133 | ±0.4834 | 3.3526 | ±0.4988 | 2.628 |
| *Phellodendron amurense* | 0.0040 | ±0.0004 | 0.0183 | ±0.0040 | 0.0086 | ±0.0038 | 0.1674 | ±0.0712 | 0.0858 | ±0.0103 | 0.0190 | ±0.0095 | 0.0241 | ±0.0124 | 0.0575 | ±0.0209 | 2.251 |
| *Skimmia japonica* | n.d. | | n.d. | | n.d. | | 3.2352 | ±0.2541 | n.d. | | 0.2189 | ±0.0151 | 8.5195 | ±0.6756 | 245.8660 | ±13.9859 | 1.395 |
| *Toddalia asiatica* | n.d. | | n.d. | | 0.0009 | ±0.0003 | 0.0268 | ±0.0037 | 0.0144 | ±0.0002 | 0.0010 | ±0.0001 | 0.0032 | ±0.0010 | 0.0049 | ±0.0021 | 1.977 |
| *Zanthoxylum ailanthoides* | 0.0865 | ±0.0038 | 0.0014 | ±0.0006 | 0.4957 | ±0.0176 | 1.4214 | ±0.1079 | 0.0230 | ±0.0070 | 0.2827 | ±0.0189 | 0.0041 | ±0.0004 | 0.0030 | ±0.0008 | 3.234 |
| *Zanthoxylum armatum* | n.d. | | n.d. | | n.d. | | n.d. | | n.d. | | n.d. | | 0.2314 | ±0.0092 | n.d. | | 0.000 |
| *Zanthoxylum piperitum* | 0.0319 | ±0.0018 | 0.0151 | ±0.0087 | 0.0044 | ±0.0012 | 0.8148 | ±0.0741 | 0.1068 | ±0.0413 | 0.0067 | ±0.0024 | 19.5814 | ±0.4789 | 0.3468 | ±0.0099 | 2.747 |
| *Zanthoxylum schinifolium* | 0.0001 | ±0.00002 | 0.0006 | ±0.0001 | 0.0006 | ±0.0002 | 0.0062 | ±0.0014 | 0.0076 | ±0.0029 | 0.0016 | ±0.0010 | 0.0005 | ±0.0003 | 0.0039 | ±0.0021 | 2.184 |

**Table S3** Host plant of each *Papilio* species and their furanocoumarin profiles. Host plants of each *Papilio* species are represented as 1 and non-hosts are shown as 0. Plant species that were chemically analyzed in this study are shown in bold.

|  | *P. dehaanii* | *P. helenus* | *P. maackii* | *P. macilentus* | *P. memnon* | *P. polytes* | *P. protenor* | *P. xuthus* | *P. macahon* |
| --- | --- | --- | --- | --- | --- | --- | --- | --- | --- |
| Host plant  Furanocoumarin amount | 33.04 | 13.28 | 86.85 | 48.61 | 1.79 | 4.40 | 30.50 | 24.52 | - |
| Host plant CCI | 1.895 | 1.969 | 2.293 | 2.071 | 1.780 | 2.172 | 1.901 | 1.840 | - |
|  |  |  |  |  |  |  |  |  |  |
| *Citrus aurantiifolia* | 0 | 0 | 0 | 0 | 0 | 0 | 0 | 1 | 0 |
| *Citrus aurantium* | 0 | 1 | 0 | 0 | 1 | 1 | 1 | 1 | 0 |
| ***Citrus depressa*** | 1 | 1 | 0 | 0 | 1 | 1 | 1 | 1 | 0 |
| *Citrus hassaku* | 0 | 1 | 0 | 0 | 1 | 1 | 0 | 1 | 0 |
| *Citrus hystrix* | 0 | 0 | 0 | 0 | 1 | 1 | 0 | 0 | 0 |
| *Citrus japonica* | 0 | 1 | 0 | 0 | 1 | 1 | 1 | 1 | 0 |
| ***Citrus junos*** | 1 | 1 | 0 | 0 | 1 | 0 | 1 | 1 | 0 |
| *Citrus kinokuni* | 1 | 0 | 0 | 0 | 1 | 0 | 1 | 1 | 0 |
| ***Citrus limon*** | 1 | 0 | 0 | 0 | 1 | 1 | 1 | 1 | 0 |
| *Citrus madurensis* | 0 | 0 | 0 | 0 | 0 | 1 | 0 | 0 | 0 |
| *Citrus maxima* | 0 | 1 | 0 | 0 | 1 | 1 | 1 | 1 | 0 |
| *Citrus mitis* | 0 | 0 | 0 | 0 | 0 | 0 | 0 | 1 | 0 |
| *Citrus natsudaidai* | 1 | 1 | 0 | 1 | 1 | 1 | 1 | 1 | 0 |
| *Citrus paradisi* | 0 | 0 | 0 | 0 | 1 | 0 | 0 | 1 | 0 |
| *Citrus reticulata* | 0 | 1 | 0 | 0 | 1 | 0 | 0 | 1 | 0 |
| *Citrus sinensis* | 0 | 0 | 0 | 0 | 0 | 0 | 0 | 1 | 0 |
| *Citrus sinensis var. brasiliensis* | 0 | 0 | 0 | 0 | 1 | 0 | 0 | 1 | 0 |
| *Citrus tachibana* | 1 | 1 | 0 | 0 | 1 | 1 | 1 | 1 | 0 |
| *Citrus tamurana* | 0 | 0 | 0 | 0 | 0 | 0 | 0 | 1 | 0 |
| *Citrus tankan* | 0 | 0 | 0 | 0 | 1 | 1 | 0 | 1 | 0 |
| ***Citrus trifoliata*** | 1 | 1 | 0 | 1 | 1 | 1 | 1 | 1 | 0 |
| ***Citrus unshiu*** | 1 | 1 | 0 | 0 | 1 | 1 | 1 | 1 | 0 |
| *Citrus x latifolia* | 0 | 0 | 0 | 0 | 0 | 0 | 0 | 1 | 0 |
| *Dictamnus albus subsp. albus* | 0 | 0 | 0 | 0 | 0 | 0 | 0 | 1 | 0 |
| *Glycosmis citrifolia* | 0 | 0 | 0 | 0 | 0 | 1 | 1 | 0 | 0 |
| ***Orixa japonica*** | 1 | 1 | 0 | 1 | 0 | 0 | 1 | 0 | 0 |
| ***Phellodendron amurense*** | 1 | 1 | 1 | 1 | 0 | 0 | 1 | 1 | 0 |
| *Ruta graveolens* | 1 | 0 | 0 | 1 | 0 | 0 | 1 | 1 | 0 |
| *Skimmia japonica var. intermedia f. repens* | 1 | 0 | 1 | 1 | 0 | 0 | 1 | 1 | 0 |
| ***Skimmia japonica var. japonica*** | 1 | 0 | 0 | 1 | 0 | 0 | 1 | 1 | 0 |
| *Skimmia japonica var. lutchuensis* | 0 | 0 | 0 | 0 | 0 | 0 | 1 | 0 | 0 |
| *Tetradium daniellii* | 0 | 1 | 0 | 0 | 0 | 0 | 1 | 0 | 0 |
| *Tetradium glabrifolium var. glaucum* | 1 | 1 | 1 | 0 | 0 | 1 | 1 | 1 | 0 |
| *Tetradium ruticarpum* | 0 | 0 | 0 | 0 | 0 | 0 | 1 | 1 | 0 |
| ***Toddalia asiatica*** | 0 | 1 | 0 | 0 | 1 | 1 | 1 | 1 | 0 |
| ***Zanthoxylum ailanthoides*** | 1 | 1 | 1 | 1 | 0 | 1 | 1 | 1 | 0 |
| *Zanthoxylum ailanthoides var. inerme* | 0 | 0 | 0 | 0 | 0 | 0 | 0 | 1 | 0 |
| ***Zanthoxylum armatum var. subtrifoliatum*** | 1 | 1 | 0 | 1 | 0 | 0 | 1 | 1 | 0 |
| *Zanthoxylum beecheyanum var. alatum* | 0 | 0 | 0 | 0 | 0 | 1 | 0 | 1 | 0 |
| *Zanthoxylum beecheyanum var. beecheyanum* | 0 | 0 | 0 | 0 | 0 | 0 | 1 | 1 | 0 |
| *Zanthoxylum fauriei* | 0 | 1 | 0 | 0 | 0 | 0 | 0 | 1 | 0 |
| *Zanthoxylum nitidum* | 0 | 1 | 0 | 0 | 1 | 1 | 1 | 1 | 0 |
| ***Zanthoxylum piperitum*** | 1 | 1 | 0 | 1 | 0 | 1 | 1 | 1 | 0 |
| *Zanthoxylum piperitum f. inerme* | 0 | 0 | 0 | 0 | 0 | 0 | 0 | 1 | 0 |
| *Zanthoxylum scandens* | 0 | 0 | 0 | 0 | 0 | 0 | 1 | 0 | 0 |
| ***Zanthoxylum schinifolium*** | 1 | 0 | 0 | 1 | 1 | 1 | 1 | 1 | 0 |
| Apiaceae | 0 | 0 | 0 | 0 | 0 | 0 | 0 | 0 | 1 |

**Table S4** Stats of transcriptome analyses

| Species | Reads | Contigs | N50 |
| --- | --- | --- | --- |
| *Papilio dehaanii* | 82,474,414 | 58,439 | 1,100 |
| *Papilio helenus* | 73,228,704 | 51,610 | 1,558 |
| *Papilio maackii* | 87,811,266 | 55,311 | 1,187 |
| *Papilio machaon* | 85,879,366 | 53,403 | 1,378 |
| *Papilio macilentus* | 73,753,764 | 42,010 | 1,284 |
| *Papilio memnon* | 74,220,296 | 52,963 | 1,492 |
| *Papilio polytes* | 80,573,528 | 48,192 | 1,097 |
| *Papilio protenor* | 78,509,746 | 41,003 | 1,341 |
| *Papilio xuthus* | 90,674,692 | 54,819 | 1,409 |

**Table S5** Results of branch site model test and positively selected sites based on rearranged and reconciled gene tree by NOTUNG.

| Branch | lnL alt | lnL null | delta L | *P* value | BEB (>0.90) |
| --- | --- | --- | --- | --- | --- |
| **1** | -23426.80 | -23429.56 | 2.76 | **0.019** | 76, 0.911 |
|  |  |  |  |  | 152, 0.965 |
|  |  |  |  |  | **217, 0.950** |
|  |  |  |  |  | **223, 0.933** |
|  |  |  |  |  | **230, 0.947** |
|  |  |  |  |  | **241, 0.939** |
|  |  |  |  |  | **262, 0.949** |
|  |  |  |  |  | 322, 0.906 |
|  |  |  |  |  | **381, 0.945** |
|  |  |  |  |  | **384, 0.945** |
|  |  |  |  |  | **417, 0.945** |
| 2 | -23440.25 | -23441.63 | 1.38 | 0.097 |  |
| **6** | -23443.80 | -23445.56 | 1.76 | 0.061 |  |
| 8 | -23442.61 | -23445.01 | 2.40 | **0.029** |  |
| 9 | -23441.00 | -23441.34 | 0.33 | 0.413 |  |
| 10 | -23442.16 | -23442.52 | 0.36 | 0.399 |  |

The branch names and sites with bold show branches or sites with signature of positive selection in the same analyses with a ML tree without reconciliation (from Table 1). The observed evidences of positive selection on Branch 1 on the ML tree are confirmed using reconciled tree but that of Branch 6 is not supported from this analysis.

lnL alt: log likelihood for alternative model which allows having unfixed dN/dS values at the branch. lnL null: log likelihood for null model with fixed dN/dS ratios. Delta L: 2(lnL alt - lnL null) for the likelihood ratio test (LRT). *P* values are from LRT. BEB analysis shows the site positions with signatures of positive selection with posterior probability (0.90 cutoff). Positions are based on Fig. S3.

**Supplementary Figures**

**Fig. S1** (a) A rearranged and reconciled *CYP6B* gene tree along with the species tree by NOTUNG. The numbered branches show conserved branches from *CYP6B* ML gene tree and these were tested for positive selection. The bold branch show signature of positive selection in both of the two branch site model tests based on the ML tree and reconciled tree. Each *Papilio* species is colored based on its phylogenetic relationships from reconstructed species tree. (b)A ML species tree of nine *Papilio* species from transcriptome data with 4 outgroups. The tree was constructed based on 567,026bp from 858 BUSCO genes. Numbers on each node show bootstrapping values from 1000 iteration.

**Fig. S2** Correlation tests between the observed *CYP6B* numbers and host plant spectrum of each *Papilio* species. There is no significant correlation between *CYP6B* numbers and host plant spectrum (*CYP6B* *vs*. CCI, *P* = 0.376, R^2^ = 0.136; *CYP6B vs*. furanocoumarin amount, *P* = 0.848, R^2^ = 0.006; *CYP6B vs*. PC1, *P* = 0.731, R^2^ = 0.021; *CYP6B* *vs*. PC2, *P* = 0.187, R^2^ = 0.270)

**Fig. S3** Amino acid sequences of *CYP6B* found in transcriptome of *Papilio* species in this study. The sequences are sorted along with the tip order in the phylogeny in Fig. 4a. Green colored sites are predicted substrate recognition sites (SRSs). The sites with blue show the most conserved motif in CYPs. Grey colored sequences are from branches with a signature of positive selection and positively selected sites are marked in red.

**Fig. S4** ML phylogeny of *CYP6B* genes (amino acid sequences) from *Papilio* species including genome data from *P. memnon* (orange) and *P. machaon* (blue). Branches with a signature of positive selection are shown in bold. Numbers on each node show bootstrapping value from 1000 iteration. Branch1 does not include genes from both *P. memnon* and *P. machaon*.
